# The Osteopathic Vertebra Put into Place

**Published:** 1907-01

**Authors:** 


### The Osteopathic Vertebra Put into Place.

JUST as these pages are being reduced to type, *The Post-Graduate,* for December, 1906, presents its appearance, and our eye falls on the following editorial paragraph relating to the demands of osteopaths for a separate state board of examiners. It is such a clear exposition of the situation that we quote it in full and commend it to those interested:

> "It is claimed in some quarters and almost intimated in "Dr. Van Fleet's article,[1] that the reason for the necessity "of one board is in the determination of the osteopaths to "secure a board for themselves. When osteopathy, like "homeopathy and electicism, comes to be a sect in medicine "embracing all the departments of medicine and surgery, it "will be time enough to talk about giving them a separate "Board of Examiners and about the injustice of not grant-"ing it. The cases are not parallel. The Homeopathic "practitioners and the Eclectics, so long as they were recog-"nised by the State with their separate State Societies, as "an organised society of practitioners, were entitled to a "Board of Examiners, but a band of confederated charlatans, "no matter how many rich and wise people consult them, "no matter how many people report the cases cured when "the regular profession had abandoned them, so long as "they have not a chartered existence with a complete system "of instruction in all departments of Medicine and Surgery, "have no right to ask the State for a separate board, no "right to complain of injustice because they haven't it. "As has been said to them over and over again, they have "only to learn the principles of anatomy, physiology, chem-"istry, and all that constitutes a medical education just as "the other sects are compelled to do, to be allowed by the "State to practise as they will with osteopathy, or Chris-"tian Science, or Chinese vegetable and animal mixtures, but "we do not believe that the Legislature after hearing the

1. Published in the Post-Graduate, October, 1906.

"case out, will ever give them a separate board. Much as "we desire the success of Dr. Van Fleet's plan, we can not "consider that there is any argument for it in the clamor of "the Osteopaths for a separate Board because each of the "three Schools of Medicine has one."

---

JOHN G. WICKSER, of Buffalo, president of the state prison commission, made a personal inspection of the Erie county jail and penitentiary on November 14, 1906. His report to the commission was made public December 6. He criticises both institutions.

The most serious of his comments is that with reference to the Erie county jail, where, he says, facilities should be provided for giving the prisoners, confined for any considerable length of time, a certain amount of open-air exercise. Prisoners awaiting trial are presumed to be innocent until proven guilty. It is a disgrace that such prisoners are sometimes held in jail for months without being allowed any outdoor exercise.

Witnesses and sometimes children have been locked up for months—even during the hot weather—without any such privilege. Even convicts in prison are uniformly given exercise in the open air.

Mr. Wickser finds also that all the male prisoners of every degree of criminality commingle in the jail corridor during their entire terms of imprisonment. The commingling of youthful offenders with hardened and professional criminals is certainly unwise, and provision should be made for their separation. The jail is without a hospital. The Sheriff is compelled to use the witness or detention room for such purpose. That compels him to keep prisoners charged with serious crimes in the same quarters with witnesses, juveniles and civil prisoners, which is also contrary to law.

This is a state of affairs that is deplorable and for which the proper authority should be held responsible. An unsanitary jail without a hospital is an abomination.

---

THE AMERICAN UROLOGICAL ASSOCIATION, second section, at a meeting held at New York, October 24, 1906, adopted a memorial on the death of Dr. William K. Otis, reported by a committee consisting of Ramon Guiteras, A. Ernest Gallant, and Ferd. C. Valentine, which reads as follows:

William Kelly Otis's earthly career ended on September 22, 1906. To the members of the American Urological Association, his death is a threefold blow. Most of us knew him intimately from his childhood; by his decease we lose a consistent friend, a charming companion, a most estimable colleague.

To the science of urology his death means an irreparable loss. Cut off in the midst of his career, his inventive genius is stopped; the new and useful instruments he was continually devising must now be perfected by other hands. The advances in our work, he can no longer aid in developing. The American Urological Association loses one of its founders, one of its most active coadjutors, one of its truest adherents.

Our association shares with the family of William K. Otis, with the profession at large, and with that world in which true manhood is understood and appreciated, that deep grief which the death of so noble a character inspires.

---

The Buffalo Academy of Medicine at a recent meeting took up the question of the proposed new system of water supply. Dr. P. W. Van Peyma introduced a resolution to the effect that, in securing an adequate water supply, it should not be overlooked that a sufficient supply of pure drinking water should be obtained. Dr. Van Peyma, in explanation, stated that he was not committed to any particular plan, but that purity should be insisted upon in whatever method or system was adopted. He thought there should be filtration either in the vicinity of Hamburg or some nearer point. Dr. W. D. Greene, health commissioner, favored the report of George W. Fuller, the expert. Dr. Ernest Wende, the incoming health commissioner, was not prepared to make a recommendation as to what should be done to secure a supply of pure drinking water, but he was quite certain that the present system was utterly deficient. He hoped the government would prevent the dumping of sewage into fresh water streams. Dr. Van Peyma's resolution was passed by a unanimous vote.

---

At the last session of the Paris Academy of Medicine, Dr. Vidal (The Tribune) called attention to the great danger of contagion from the use of Oriental carpets. These carpets come from countries in which dysentery and other diseases prevail. The disease germs settle in the fibres of the material, and their transmission to the user is a probability if the textiles are not carefully disinfected. Dr. Vidal told of two cases that illustrate the danger. They occurred recently in Paris. An elderly man, a collector of rugs, received a dealer who exhibited to him a number of Oriental samples. He finally bought two Japanese tapestries, upon which the purchaser's three-year-old child played for a while. Eight days later the child died, having been infected with dysentery. A few days later the father also had an attack of the same disease that resulted in death.

---

The recent police order in New York against permitting children to use the roadways or streets as playgrounds emphasizes the desirability of increasing the number of recreation centres and public playgrounds. A number of small playgrounds, judiciously scattered over the city would be of incalculable benefit to the children, not only for their pleasure, but also for their health and morals and would also be a boon to many adults who are greatly annoyed and embarrassed in their legitimate use of the streets by the throngs of children who now make the passage of vehicles slow and difficult.

This suggestion, taken from The Tribune, applies with equal force to Buffalo.

---

ANNOUNCEMENT was made from Philadelphia, December 24, 1906, that Andrew Carnegie had given $100,000 to the College of Physicians of that city to build a new library in twenty-second street, on condition that an equal amount be raised by the society. All but $20,000 of the amount has been raised, and the best known surgeons and practising physicians in the city are working untiringly to get the remainder of the money. It is the oldest medical society in this country, having been established in 1787. It bears the same relation to Philadelphia and vicinity that the New York Academy of Medicine does to greater New York and the surrounding country.

---

As announced from Stockholm, the Nobel prize of 200,000 francs for medicine has been awarded to Professor Camillo Golgi, of the University of Pavia. Professor Golgi distinguished himself by his works on the changes of the lymphatic vessels of the brain and his discovery relating to malarial fever. The scientist was born at Corteno, July 7, 1844, and since 1876 has taught at the University of Pavia, where he was graduated. Another Nobel prize is to be awarded to the Spanish scientist Cazal.

